# Synthesis of a Zinc Oxide Nanoflower Photocatalyst from Sea Buckthorn Fruit for Degradation of Industrial Dyes in Wastewater Treatment

**DOI:** 10.3390/nano9121692

**Published:** 2019-11-26

**Authors:** Esrat Jahan Rupa, Lalitha Kaliraj, Suleman Abid, Deok-Chun Yang, Seok-Kyu Jung

**Affiliations:** 1 Department of Oriental Medicinal Biotechnology, College of Life Sciences, Kyung Hee University Giheung-gu Yongin-si, Gyeonggi-do, Yongin 446-701, Korea; eshratrupa91@gmail.com; 2 Graduate School of Biotechnology and Ginseng Bank, College of Life Sciences, Kyung Hee University, Yongin 446-701, Korea; lalithakr95@gmail.com (L.K.); sulmanabid1994@gmail.com (S.A.)

**Keywords:** sea buckthorn fruit, zinc oxide nanoflower, dye degradation, photocatalysis, UV illumination, Congo red, malachite green, eosin Y, methylene blue

## Abstract

Green synthesis of ZnO nanoparticles has attracted research attention as a sustainable method of avoiding the destructive effect of chemicals. We synthesized a flower-shaped zinc oxide (ZnO) nanoflower (NF) from sea buckthorn fruit (SBT) by co-precipitation and characterized it using X-ray powder diffraction (XRD), X-ray photo electronic microscopy (XPS), photoluminescence (PL), field emission transmission electron microscopy (FE-TEM), and Fourier-transform infrared (FT-IR) spectroscopy. The ability of the ZnO/NF to degrade cationic and anionic dyes, including malachite green (MG), Congo red (CR), methylene blue (MB), and eosin Y (EY), under ultraviolet illumination was studied. The photocatalyst degraded approximately 99% of the MG, MB, CR and EY dyes within 70, 70, 80, and 90 min of contact time, respectively, at a dye concentration of 15 mg/L, 5 mg/L, SBT-ZnO/NF degraded 100% of the MG, MB, CR and EY dyes within 23, 25, 28, and 30 min, respectively. The results indicate that SBT-ZnO/NFs as synthesized is an inexpensive, non-toxic, rapid, and reusable photocatalyst that can play an enhanced role in wastewater treatment.

## 1. Introduction

Demand for chemically synthesized dyes and other materials is increasing with growing populations. As byproducts of the chemical, textile, and leather industries, dyes are often associated with toxic chemicals that are carcinogenic to humans, as well as toxic to microbes that play key ecological roles [1]. Much research effort has therefore focused on developing tools to remove hazardous dye chemicals from the environment [2]. Micro-filtration, adsorption, ultrafiltration, and ion exchange are among the well-known tools for addressing this problem, but all impose some negative environmental impact [3,4]. Industrial anionic dyes are difficult to remove from water because of the presence of negative ions [5,6,7]. Nanotechnologies are widely assumed to offer promising avenues to tackle such challenges due to their high surface-area-to-volume ratios. Metal nanoparticles with a strong oxidizing radical (OH^.^), such as zinc oxide (ZnO), titanium oxide, and tungsten oxide, have already proven to be powerful photocatalysts [8]. ZnO in particular is known for its low cost, water de-toxification abilities, high reaction and mineralization rates, and large number of active sites that enhance degradation of dyes under ultraviolet (UV) illumination [9,10,11]. ZnO nanoparticles (NPs) have attracted research attention due to their low cost, low environmental impact, and rapid degradation of toxic dyes from wastewater. ZnO nanoparticles can be synthesized using various chemical methods, including those that use environmentally benign source materials instead of harmful chemicals and surfactants. Various reports have shown that the crystalline structure of ZnO nanoparticles from plant extracts is associated with efficient dye degradation [12]. Here, we prepared crystalline-shaped ZnO nanoflowers (NFs) using an active compound found in sea buckthorn (SBT) fruit to produce crystalline nanoparticles that may enhance the degradation rate of toxic dyes. *Hippophae rhamnoides,* commonly known as the vitamin tree or sea buckthorn, is a well-known shrub of the Elaeagnaceae family. Sea buckthorn fruit contain many bioactive compounds, such as flavonoids, unsaturated fatty acids, phenolic compounds, phytosterols, carotenoids, and tocopherol [12,13,14,15], and exhibit antioxidant, cell-proliferation, antibacterial, and anticancer properties [16,17,18,19]. Sea buckthorn ZnO nanoflowers (SBT-ZnO/NFs) have been used as green nano catalysts because of their crystallinity, shape, high dye-degradation efficiency (99%), and rapid action (<1 h). To evaluate dye-degradation efficiency, malachite green (MG), methylene blue (MB), Congo red (CR) and Eosin Y (EY) were chosen as model dyes. Our results suggest that SBT-ZnO/NFs can serve as safe, low-cost, and biodegradable materials for removal of hazardous dyes from wastewater.

## 2. Materials and Methods

### 2.1. Materials

Samples of *Hippophae rhamnoides* (sea buckthorn) fruit were collected from a local market in China. Zinc nitrate hexahydrate (> 98%) was purchased from Sigma–Aldrich, USA. Analytical grade NaOH was purchased from Dae Jung Chemicals and Metals Co. Ltd. (Pyeontaek, Korea). All dyes were obtained from Sigma–Aldrich, USA, and all chemicals were used without further purification.

### 2.2. Preparation of Sea Buckthorn Fruit Extract

Fresh sea buckthorn fruit samples were washed repeatedly with water and dried using an air drier. The dried samples were crushed into powder, 5 g of which was mixed with 100 mL of water. Phytochemicals were extracted from the powdered fruit using a high-pressure autoclave at 100 °C for 1 h. Autoclaved extracts were filtered using Whatman no. 1, 110 mm filter paper. The extracts were then centrifuged at 5000 rpm for 10 min to remove undesirable solids.

### 2.3. Synthesis of Zinc Oxide Nanoflowers from Sea Buckthorn Fruit Extract

**Figure 1:**
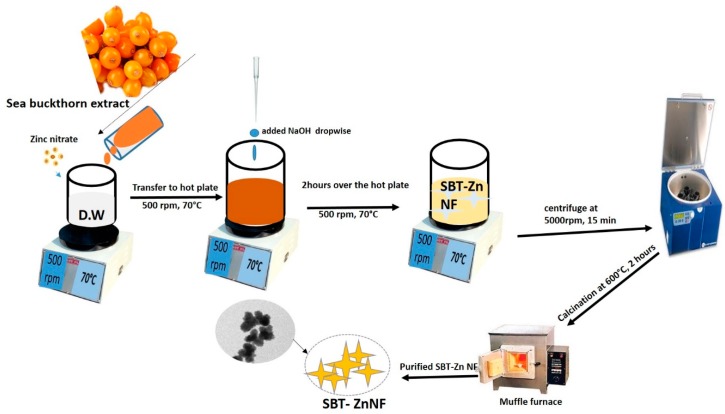
Synthesis sea buckthorn fruit—zinc oxide nanoflowers (SBT-ZnO/NF) by co-precipitation.

The SBT-ZnO/NFs were synthesized by co-precipitation using a previously described method with minor modifications [20]. Zinc nitrate hexahydrate and NaOH were used as precursors, and SBT fruit extract was used as a reducing and coating agent. To synthesize SBT-ZnO/NFs, 20% of the SBT fruit extract was collected and continuously stirred. Approximately 10 mL of 0.1 M zinc nitrate salt was added dropwise to the homogeneous mixture, which was then transferred to a hot plate. When the temperature of the mixture reached 70 °C, 15 mL of 0.2 M NaOH was added dropwise to the walls of the flask with continuous stirring. The color changed from yellow to milky white, which indicated formation of SBT-ZnO/NFs. The synthesized materials were kept on the hot plate for up to 2 h to complete the reaction. The SBT-ZnO/NF mixture was washed with distilled water at least three times to remove unreacted materials and centrifuged at 5000 rpm for 15 min. Finally, the SBT-ZnO/NFs were collected as a solid material and calcinated in a muffle furnace for 3 h. The process is illustrated in Figure 1.

### 2.4. Characterization of SBT-ZnO/NFs

The synthesized NPs were characterized by instrumental analysis. Bio-reduction of metallic nanoparticles from metal ions was confirmed through analytical methods. The reaction mixtures’ absorption spectra at 300–800 nm was produced using a UV-visible light spectrophotometer (Ultrospec 2100, pro-USA) and a 10-nm-long quartz cuvette. Field emission transmission electron microscopy (FE-TEM; JEM-2100 F, JEOL, USA) was conducted at an operating voltage of 200 kV. The selected area electron diffraction (SAED) pattern supplied the crystallinity or amorphous condition pattern of the NF. Energy-dispersive X-ray spectroscopy (EDX) supplied purity data on the NFs, and elemental mapping provided the distribution of the particles inside each NF. The SBT-ZnO/NF droplets were placed onto a carbon-coated copper grid, dried in an oven at 60 °C, and transferred to the analyzer. X-ray powder diffraction (XRD; D8 Advance, Bruker, Germany) confirmed crystallinity of SBT-ZnO/NFs in a 2θ range of 20°–80° with Cu-Kα radiation of 1.54 Å, an operating voltage of 40 kV, and a current of 40 mA. Fourier-transform infrared spectroscopy (FT-IR) was used to analyze the functional groups inside the NFs with a Perkin Elmer spectrum at 400–450 cm^−1^. X-ray photoelectron microscopy (XPS; Thermo Electron/K-Alpha, Thermo Scientific, Seoul, Korea) was used to analyze the elemental state and composition of the nanostructures. Photoluminescence (PL) properties were analyzed using a Sinco Floormate FS-2 spectrofluorometer.

### 2.5. Photocatalytic Activity of SBT-ZnO/NFs

A 15 mg L^−1^ concentration of each dye (MG, MB, EY, and CR) was used. The chemical formula and maximum absorption of the dyes are supplied in Table 1.

The dye and catalyst were sonicated for approximately 30 min to ensure thorough mixing. To reach an adsorption–desorption equilibrium, dye and catalyst (SBT-ZnO/NF) were mixed and kept in a dark place. The reaction mixture was irradiated by a UV cross-linker (C−1000 Ultraviolet cross-linker, energy × 100 µj/cm) for approximately 10, 20, 30, 50, 60, 70, 80, and 90 min. The degradation efficient was calculated using Equation (1):

Degradation efficiency (%) *n* = (*C*_o_ − *C*_t_)/*C*_o_ (1)

where *C*_t_ is the concentration at time t, and *C*_o_ is the initial concentration at time t_0_.

## 3. Results and Discussion

### 3.1. UV-Visible Spectrophotometer Analysis

**Figure 2:**
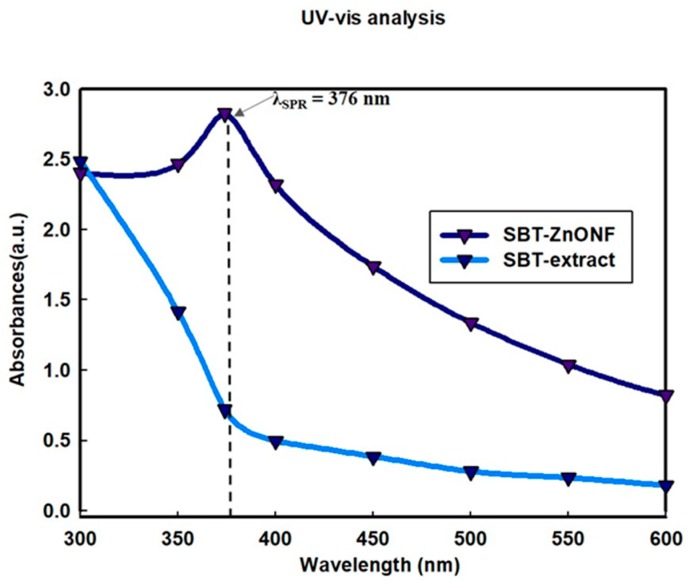
UV-visible spectroscopy of sea buckthorn (SBT) fruit extract and an absorption peak of SBT-ZnO/NFs at 376 nm.

The formation of ZnO was confirmed by UV–vis spectroscopy. The SBT-ZnO/NF exhibited surface Plasmon resonance (SPR) as a result of collective oscillation of electrons of ZnO NPs and its interaction with certain wavelengths of light. The UV-visible spectrophotometer detected a maximum absorbance peak of SBT-ZnO/NF at 376 nm, using the wavelength range 300–800 nm indicating formation of SBT-ZnO/NFs from SBT fruit extract. However, the SBT fruit extract did not show an absorption peak in the region of ZnO/NF formation, as shown in Figure 2.

### 3.2. FE-TEM Analysis

**Figure 3:**
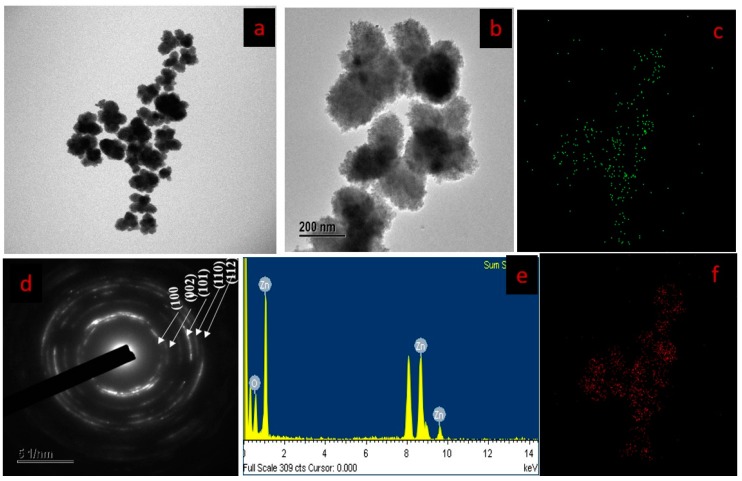
FE-TEM image of SBT-ZnO/NFs (**a**,**b**), elemental distribution of SBT-ZnO/NFs (**c**,**f**), selected area electron diffraction (SAED) pattern of SBT-ZnO/NFs (**d**), and EDX results of SBT-ZnO/NF (**e**).

The morphology of synthesized SBT-ZnO/NFs is shown in Figure 3a,b using FE-TEM analysis. The nanoflowers formation were observed; they consist of a combination of 3–4 broad petal-like structures. In Figure 3a,b, good dispersion of the nanoparticles was observed because the fruit extract has a capping effect that prevents agglomeration of nanoparticles; on the other hand ZNO prepared without SBT extract showed poor nano structure with agglomeration of nanoparticles (Figure S1). An SAED pattern revealed the high crystallinity of SBT-ZnO/NFs (Figure 3d). The purity of SBT-ZnO/NFs was examined by EDX spectroscopy, as shown in Figure 3e. Elemental mapping revealed the distribution of zinc (red dot) and oxygen (green dot) atoms through the SBT-ZnO/NFs (Figure 3c,f); and the weight and atomic percentages were approximately 79.12 (zinc), 20.88 (oxygen) and 48.11 at.% (zinc), 51.89 at.% (oxygen) respectively.

### 3.3. XRD Analysis

**Figure 4:**
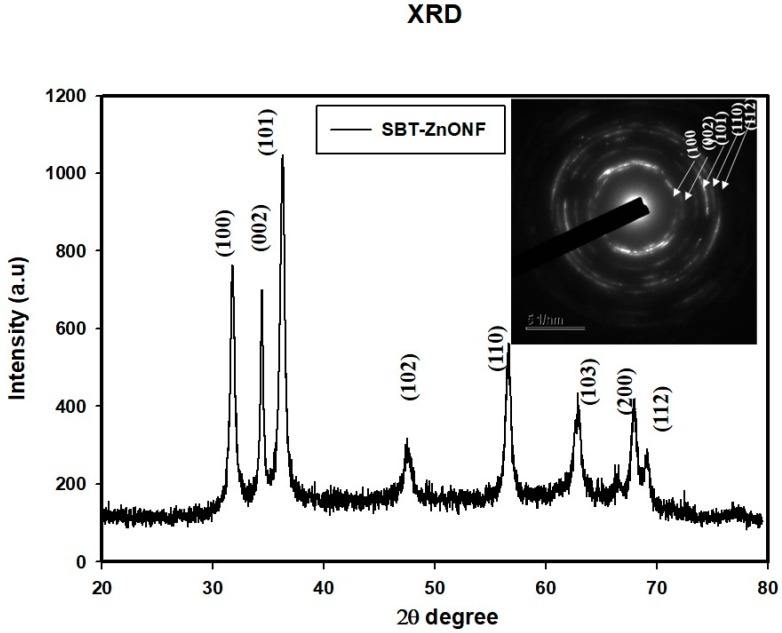
X-ray diffraction pattern of SBT-ZnO/NF and the SAED pattern.

The XRD pattern in Figure 4 depicts the crystalline structure of NFs at 2θ values of 31.82, 34.41, 36.27, 47.55, 56.61, 62.82, and 67.97, which were indexed according to Miller indices (*h*, *l*, *k*) respectively at (100), (002), (101), (102), (110), (103), and (112) in a lattice plane. These value matched well with the standard values of JCPDS NO. 36−145 and the most intense peak at 36.27 belongs to the (101) orientation, and the position of the reflection peaks of SBT-ZnO/NF indicated a hexagonal wurtzite structure. The larger crystalline size is specified by the intense peak of the pattern. The FWHM results revealed from *d*_(100)_ = 2.81 A°, *d*_(002)_ = 2.60 A°, *d*_(101)_ = 2.47 A° value with sharp and intense peaks were used to determine the size of SBT-ZnO/NF.

The nanocrystalline size calculated using the Debye–Scherrer equation was approximately 17.12 nm, Avarage of 1st 3 value. as noted in Table 2.

### 3.4. FT-IR Analysis

**Figure 5:**
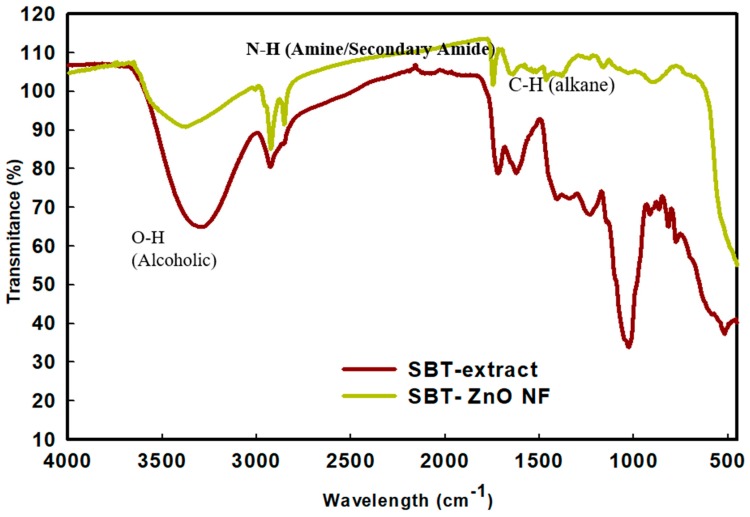
Fourier-transform infrared spectroscopic (FT-IR) peaks of SBT extract and SBT-ZnO/NFs in transmission mode.

Fourier-transform infrared (FT-IR) spectroscopic analysis was used to diagnose the phytochemicals responsible for the reduction of Zn^2+^ and stabilization of ZnO NPs. FT-IR analysis (Figure 5) revealed the spectra of the SBT extract before and after synthesis into SBT-ZnO/NFs. The Zn-O metal bond formation was confirmed by an absorption peak at 440.48 cm^−1^ [21]. However, the extract of the fruit showed no absorption peak in the area of 400–500 cm^−1^. The SBT fruit extract showed an absorption peak at 899.71 cm^−1^, which shifted to the ZnO/NF absorption peak at 1026.78 cm^−1^, indicating a =CH-H (alkene) group. The extract showed an absorption peak at 1618.09 and 1718.33 cm^−1^, which shifted to 1384.98 cm^−1^ and 1635.19 cm^−1^, indicating alkene (C-H) and ketone (C=O) groups, respectively [22]. The NFs and extract both showed absorption peaks at 2925.19 cm^−1^, which indicated presence of an N-H (secondary amine) group [23]. The absorption peak at 3385.75 cm^−1^ for the extract indicated presence of an (-OH) alcohol group. In SBT-ZnO/NFs, this peak shifted to 3423.88 cm^−1^ but still indicated an –OH group [24]. This (O-H) and (N-H) bond were due to absorbed polyphenol from the fruit extract during formation of NF. The FT-IR spectrum SBT-ZnO/NF shows prominent capping of phytochemical on the surface of the Nano-flower.

### 3.5. Photoluminescence

**Figure 6:**
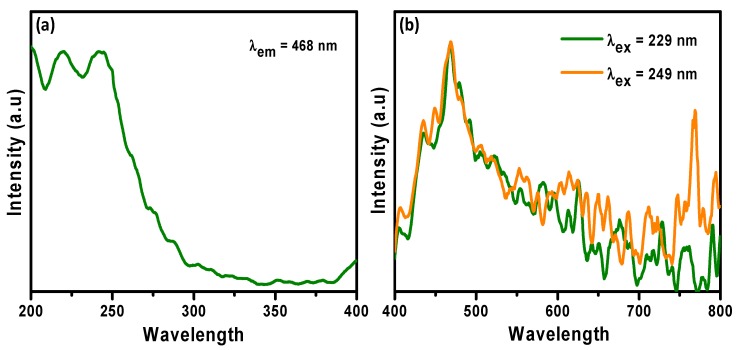
Photoluminescence spectra of SBT-ZnO/NFs. (**a**). Emission wavelength at 468 nm (**b**). Excitation wavelength at 229 nm and 249 nm of SBT-ZnO/NFs.

The photoluminescence (PL) properties of SBT-ZnO/NF are shown in Figure 6. The PL excitation spectra of SBT-ZnO/NFs were measured at an emission wavelength of 468 nm, exhibiting major peaks at 229 and 249 nm. The emission spectra at the two excitation wavelengths showed different intensities. The PL emission spectra showed intense peaks at 407, 434, 448, and 469 nm, ascribed to the deep-level effect. The remaining transitions in the longer-wavelength region are due to recombination of photo-generated holes with the singly ionized charge state of this effect. The strong UV emission corresponds to the near-band edge emission of the wide bandgap of ZnO due to annihilation of excitations. The strong PL properties revealed the high purity and superior crystallinity of the ZnO NPs.

### 3.6. X-ray Photoelectron Spectroscopy (XPS) Analysis

**Figure 7:**
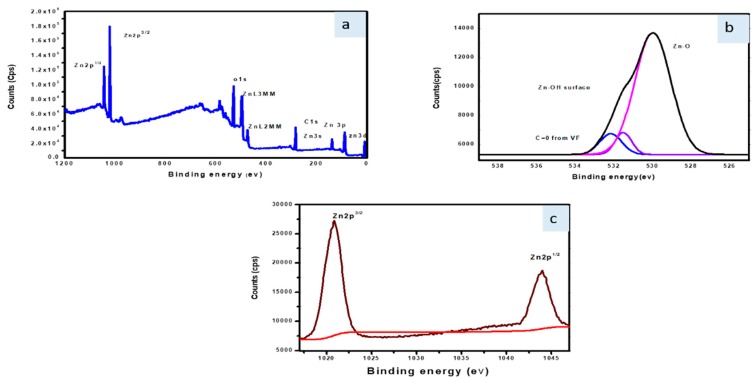
X-ray photoelectron spectroscopy. (**a**) Full-survey spectrum of SBT-ZnO/NF; (**b**) O1s binding energy area; (**c**) Zn2p binding energy region.

XPS detected Zn, C, and O peaks in the SBT-ZnO/NF structures, as shown in the wide-survey spectra (Figure 7a). During synthesis of SBT-ZnO/NFs, the carbon absorbed on the surface is related to the detected carbon-binding energy at 285 eV of the synthesis condition. In Figure 7b, the Zn2p spectrum for SBT-ZnO/NFs showed binding energies at 1021.2 and 1044.3 for Zn2p3/2 and Zn2p1/2, respectively, indicating the +2 state of Zn [25]. The binding energies at 532.9, 530.7, and 529 eV in Figure 7c are associated with C=O absorbed from SBT fruit, Zn-OH (surface oxygen), and ZnO metal bonds, respectively. The identified element inside the structure of SBT-ZnO/NFs confirmed formation of NFs through XPS analysis.

### 3.7. Photocatalytic Activity of SBT-ZnO/NF under UV Illumination

**Figure 8:**
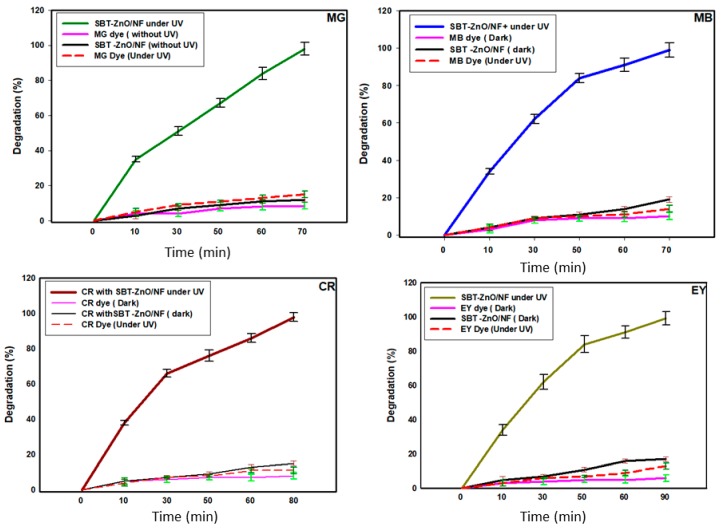
Malachite green (MG), methylene blue (MB), Congo red (CR) and eosin Y (EY) dye degradation at a high starting concentration (15 mg/L) using SBT-ZnO/NFs.

**Figure 9:**
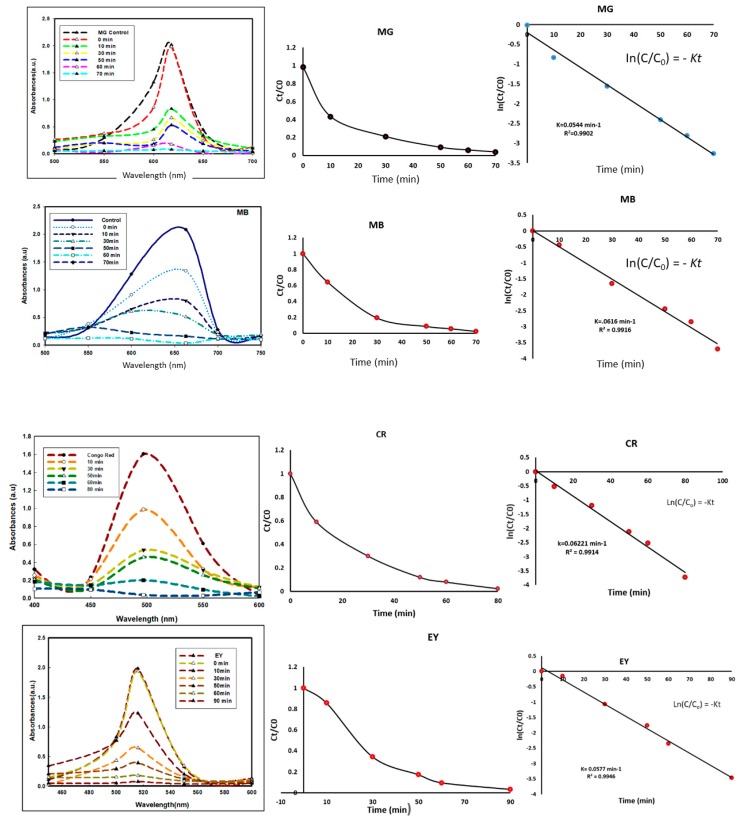
Photocatalytic activity curve of SBT-ZnO/NFs with MG, MB, CR, and EY dye degradation according to time under UV irradiation and kinetic study.

The SBT-ZnO/NF was used as a photocatalyst. Here, four types of industrial dyes were used at a concentration of 15 mg/L under UV illumination. Degradation efficacy was monitored under dark and under UV with and without catalyst, is shown Figure 8. The results suggested that only SBT-ZnO/NF showed significant degradation under UV illumination, whereas other conditions exhibited negligible degradation. All dyes (malachite green (MG), methylene blue (MB), Congo red (CR) and eosin Y (EY)) were monitored under UV illumination from a C-1000 ultraviolet cross-linker, energy × 100 µj/cm (364 nm) using SBT-ZnO/NFs at different time intervals. MG, MB, CR and EY dyes were degraded by up to 99% within 70, 70, 80, and 90 min of contact time, respectively. MG, MB, CR and EY showed absorbance peaks at 663, 618, 497 and 517 nm respectively. The intensities of peaks were decreased with respect to the time during the degradation of dye molecules (Figure 9).

**Figure 10:**
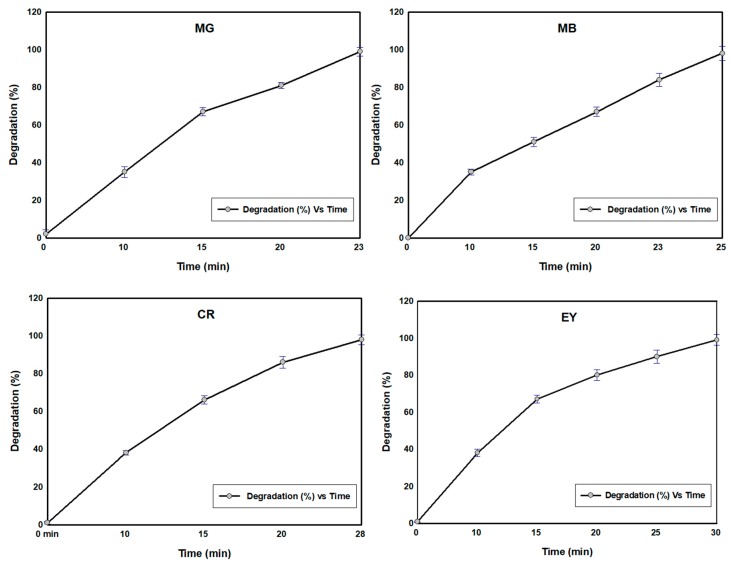
Degradation curves of SBT-ZnO/NFs over time at a low concentration (5 mg/L) of dye.

Kinetics study for all the four dyes follow the 1st order kinetics model which was confirmed from linear regression (R^2^ > 0.99) (Figure 9). There was a slight blue shift (shifted to higher frequency) in absorption spectra of all the dye molecules during degradation with respect to the time. That was in accordance with the previous study [26] and suggested that the blue shift is evidence for progressive degradation that occurred by removal of organic dyes [27]. For comparison, the dye degradation was also examined using ZnO-NPs and SBT fruit extract and the results were shown in Figure S2. SBT extract showed about 20% of dye degradation in all cases, whereas ZnO-NPs showed 60–70% efficiency. But SBT-ZnO/NF showed 100% degradation efficiency for MG, MB, CR and EY at 70, 70, 80, and 90 min, respectively. The higher degradation efficiency of SBT-ZnO/NF may be occurring due to the synergistic effect of the capping layer of phenolic compound (from SBT fruits) over the NF and the active sites of ZnO particles. SBT-ZnO/NF produced OH^.^ (hydroxyl free radical) and O^.2^ (superoxide) molecules which are mainly responsible for dye degradation. At the lower concentration (5 mg/L) of dye using the SBT-ZnO/NF catalyst, we observed that MG, MB, CR and EY dyes were degraded by 100% within 23, 25, 28, and 30 min of contact time, respectively (Figure 10).

**Figure 11:**
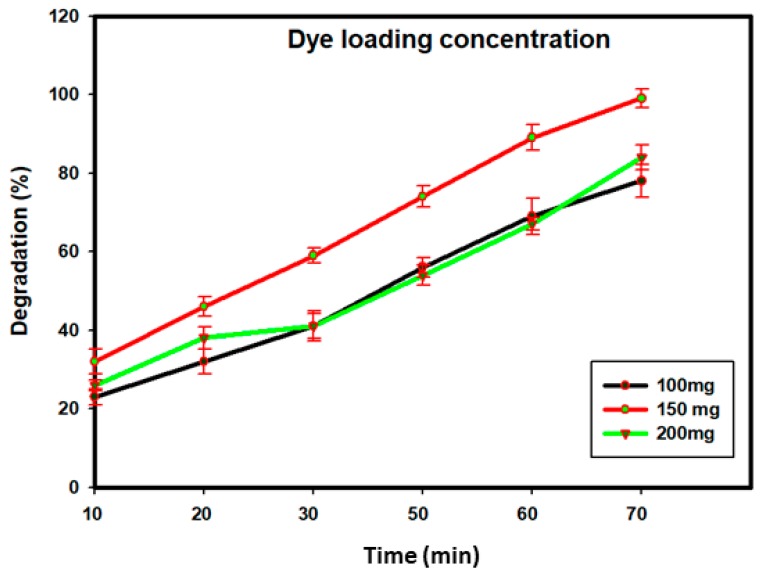
Optimization of loaded dye concentration.

As a catalyst, SBT-ZnO/NFs have the potential to decompose several dye molecules in an advanced oxidation process. Catalyst concentration is an important parameter for assessing degradation activity. In this study, we measured different concentrations (100 mg/L, 150 mg/L, and 200 mg/L) of SBT-ZnO/NFs, where the highest catalytic activity was shown at 150 mg/L of MG, as in Figure 11. The results suggest that high catalyst load can increase catalytic activity by creating more active areas. However, use of more than 150 mg of catalyst may result in overlapping peaks and disturbance to UV rays at the surface of the semiconductor, decreasing the degradation rate. Additionally, SBT-ZnO/NFs showed superior photocatalytic activity to other plant extracts reported in previous reports (Table 3).

### 3.8. Degradation 

Ultraviolet illumination causes SBT-ZnO/NF nanocatalyst surfaces to act as a semiconductor to produce photoexcitation (as described in the PL study) inside the SBT-ZnO/NF, generating lone-pair electrons and holes. The holes react with water molecules to form hydroxyl free radicals, which are responsible for degradation of the dye [34]. A possible chemical reaction is shown below.

SBT-ZnO/NF + hv → e^−^ + h^+^ + SBT-ZnO/NF, (2)

Oxidation reaction on the surface of the catalyst (SBT-ZnO/NF) can be described by:

h^+^(VB) + H_2_O → H^+^ + •OH, (3)

2h^+^ + 2H_2_O → 2H^+^ + 2H_2_O_2_, (4)

However, the lone-pair electron reacts with oxygen molecules to form superoxide molecules, which react with hydrogen ions to form hydroxyl free radicals. Ultimately, this molecule degrades the dye through a reduction process:

e^•^(CB) + O_2_ → •O_2_^−^ (5)

The oxidation- and reduction-reaction products (•OH, •O_2_^−^) in the form of free radicals both help oxidize the harmful dye to form a harmless compound, with CO_2_ and H_2_O as byproducts.

Dye molecules + (•OH, •O_2_^−^) → CO_2_ + H_2_O + byproducts (6)

**Figure 12:**
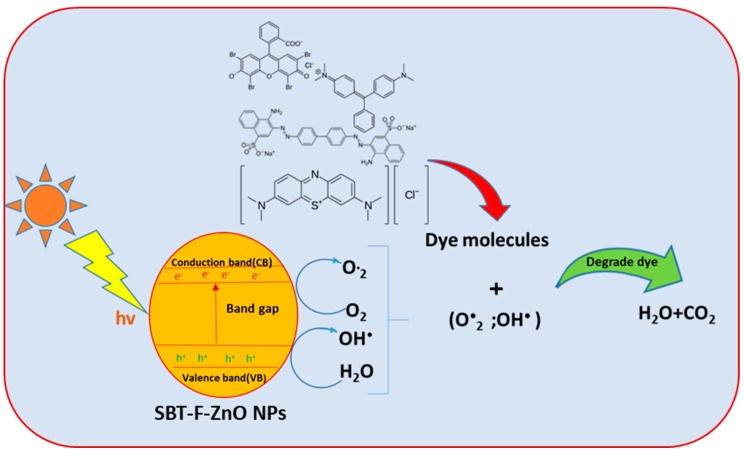
Dye degradation scheme of SBT-ZnO/NFs.

The phytochemicals inside the SBT fruit, along with the carbon traced in the XPS analysis, could help generate more OH• on the semiconductor surface. Consequently, SBT-ZnO/NFs exhibit photocatalytic activity within a short time. The full mechanism is illustrated in Figure 12.

### 3.9. Catalyst (SBT-ZnO/NF) Reusability

**Figure 13:**
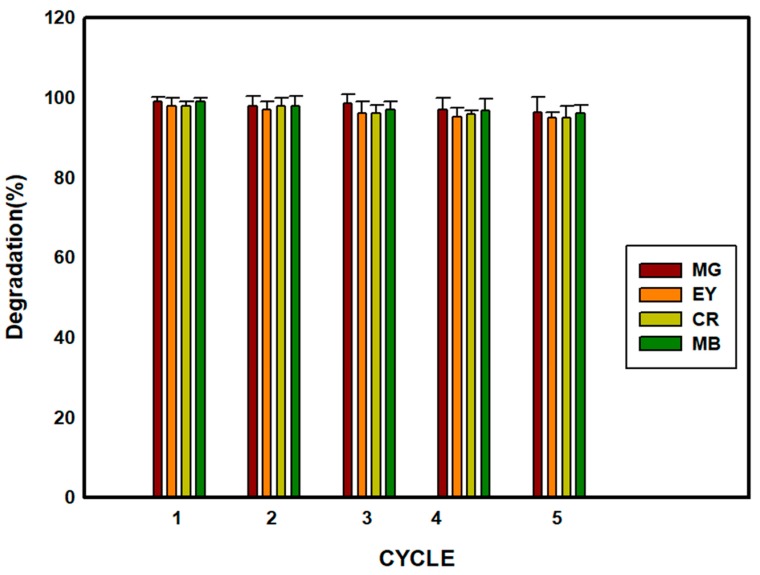
Test of the reusability of SBT-ZnO/NFs using 15 mg/L dye (MG, MB, EY, CR).

For any catalyst, reusability is an important parameter. In this study, SBT-ZnO/NF was collected via centrifugation, washed with ethanol to remove the attached substances, and dried in an oven at 60 ℃. Single reuse of this catalyst produced the same level of dye reduction; after 2–3 cycles, dye-degradation efficacy decreased slightly due to centrifugation, and self-photosensitized decomposition of dye molecules occurring in the presence of ZnO/NF, as shown in Figure 13. This suggests that SBT-ZnO/NF nanocatalysts can be used more than five times, with only a slight decline in degradation rate.

## 4. Conclusions

In summary, this study establishes an eco-friendly, greener method for the synthesis of SBT-ZnO/NF from sea buckthorn fruit extract as a capping and stabilizing agent. This NF was inspected for different dyes (MB, MG, CR, EY) degradation activity under UV illumination. The hexagonal wurtzite structure with an average size 17.15 nm was confirmed by XRD analysis. The nanoflower structure was confirmed through FE-TEM images. Elemental state and chemical composition were analyzed through XPS and EDX analysis, during which no impurities were found. The functional group which is responsible for flower-shaped NF were identified using FT-IR. PL study also indicated the generation of electron-hole pairs in the catalyst surface. The SBT-ZnO/NF exhibited >99% of degradation efficiency at 15 mg/L and 5 mg/L of all four dyes. MG dye degraded within 70 and 23 min, MB dye degraded within 70 and 25 min, CR dye degraded 80 and 28 min and EY dye degraded 90 and 30 min of contact time, respectively, for these two dye concentrations. This research highly recommends the use of SBT-ZnO/NF as a future nano catalyst for the degradation of hazardous dyes during wastewater treatment.

## Figures and Tables

**Table 1:** The structures and maximum intensity measures of dyes.

Structures of Dye Molecules	Maximum Intensity (nm)
Malachite green 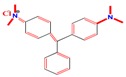	618 nm
Methylene blue 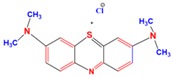	663 nm
Congo red 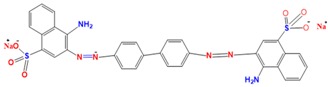	497 nm
Eosin Y 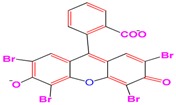	517 nm

**Table 2:** Size analysis of SBT-ZnO/NFs using XRD.

Peak No	Peak Position at (2*θ*)	d Spacing Value (A°)	FWHM Value (2*θ*)	Size (nm)	Average (nm)
100	31.82	d = 2.81163	0.5885	15.56	
002	34.412	d = 2.60226	0.3923	21.07	
101	36.272	d = 2.47333	0.5654	14.72	
102	47.552	d = 1.91472	1.2555	6.67	17.12
110	56.613	d = 1.90877	0.7454	11.63	
103	62.883	d = 1.62367	1.079	8.38	
112	67.979	d = 1.61797	1.4749	6.32	

**Table 3:** Comparison study of dye degradation for synthesized ZnO NPs from various plant sources.

Plant Extract	Dye	Dye Concentration	Catalyst Loaded	Degradation (%)	Time (min)	Reference
*Tabernaemontana divaricata*	Methylene blue	5 mg/L	100 mg	90%	90 min	[28]
*Suaeda japonica* Makino	Methylene blue	10 mg/L	50 mg	54%	180min	[29]
*Kalopanax septemlobus*	Methylene blue	10 mg/L	150 mg	97%	30 min	[30]
*Hippophae rhamnoides* leaves	Malachite green	10 mg/L	150 mg	89%	180 min	[31]
*Rubus coreanus*	Malachite green	10 mg/L	50 mg	90%	240 min	[32]
*Abelmoschus esculentus*	Methylene blue	9.5 mg/L	125 mg	100%	60 min	[33]
*Hippophoe Rhamnoides* fruits	Methylene blue	15 mg/L	150 mg	99%	70 min	This work
*Hippophoe Rhamnoides* fruits	Malachite green	15 mg/L	150 mg	100%	70 min	This work
*Hippophoe Rhamnoides* fruits	Congo red	15 mg/L	150 mg	99%	80 min	This work
*Hippophoe Rhamnoides fruits*	Eosin Y	15 mg/L	150 mg	100%	90 min	This work

## Supplementary Materials

The following are available online at https://www.mdpi.com/2079-4991/9/12/1692/s1, Figure S1: Comparison images for ZnO-NPs (**a**) and SBT-ZnO/NF (**b**), Figure S2: Comparison study for degradation (%) of (MB, MG, CR, EY) dyes using SBT-ZnO/NF, SBT-Ext and ZnO NPs (prepared in chemical method) catalyst under UV illumination.
