# Supplementary material for: Synthesis of a Zinc Oxide Nanoflower Photocatalyst from Sea Buckthorn Fruit for Degradation of Industrial Dyes in Wastewater Treatment
Source: Nanomaterials (Basel). 2019 Nov 26;9(12):1692. doi: 10.3390/nano9121692 (PMC6970228; doi:10.3390/nano9121692)
Supplement: Supplementary file 1 [file nanomaterials-09-01692-s001.pdf]

Supporting information

Synthesis of a zinc oxide nanoflower photocatalyst from sea buckthorn fruit for degradation of industrial dyes in wastewater treatment

Esrat Jahan Rupa,[1] Lalitha Kaliraj,[2] Suleman Abid,[2] Deok-Chun Yang,[1,2*] Seok-Kyu Jung[1,2*]

1 Department of Oriental Medicinal Biotechnology, College of Life Sciences, Kyung Hee University Giheung-gu Yongin-si, Gyeonggi-do, Republic of Korea; khwb6000@khu.ac.kr

2 Graduate School of Biotechnology and Ginseng Bank, College of Life Sciences, Kyung Hee University, Yongin, 446-701, Republic of Korea; khsa0035@khu.ac.kr

* Correspondence: dcyang@khu.ac.kr; Tel.: +82-10-4055-2779 (D.C.Y) & gentlemen71@msn.com;

Tel.: +82-10-4055-2779

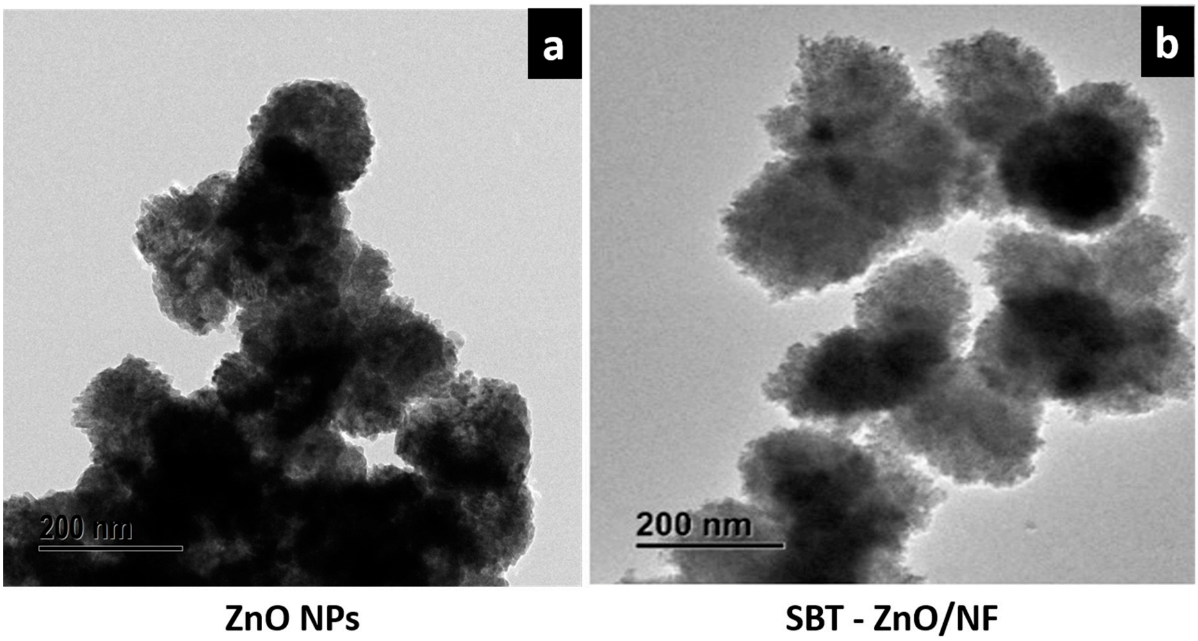

Figure S1. Comparison images for ZnO-NPs (a) and SBT-ZnO/NF (b)

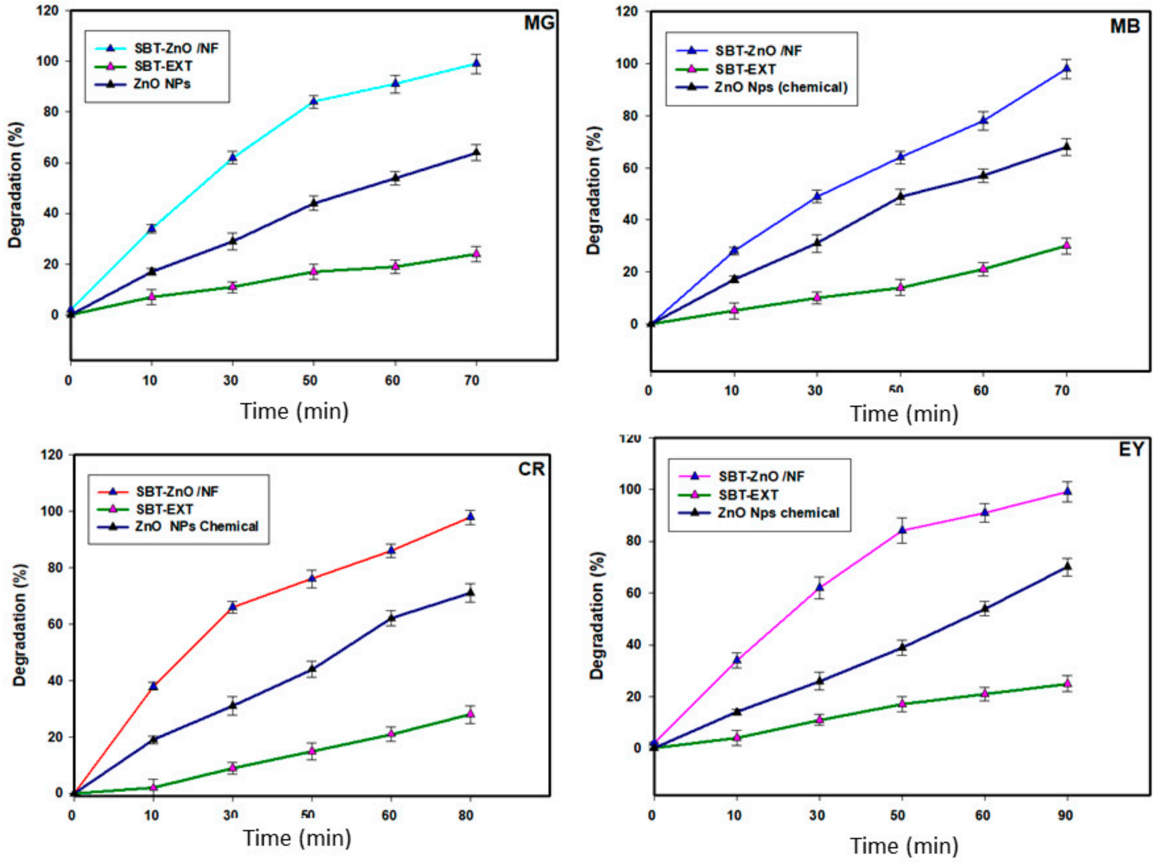

Figure S2. Comparison study for degradation (%) of (MB, MG, CR, EY) dyes using SBT-ZnO/NF, SBT-Ext and ZnO NPs (prepared in chemical method) catalyst under UV illumination.
